# Strukturierung des Notfallmanagements in Pflegeheimen: Ergebnisse interprofessioneller Fokusgruppeninterviews

**DOI:** 10.1007/s00391-021-01958-9

**Published:** 2021-08-13

**Authors:** Sven Schwabe, Jutta Bleidorn, Andreas Günther, Olaf Krause, Nils Schneider, Juliane Poeck

**Affiliations:** 1 Institut für Allgemeinmedizin und Palliativmedizin, Medizinische Hochschule Hannover, Carl-Neuberg-Str. 1, 30625 Hannover, Deutschland; 2 Institut für Allgemeinmedizin, Universitätsklinikum Jena, Jena, Deutschland; 3 Fachbereich Feuerwehr, Stadt Braunschweig, Braunschweig, Deutschland; 4 Zentrum für Medizin im Alter, Diakovere Henriettenstift Hannover, Hannover, Deutschland

**Keywords:** Pflegeeinrichtungen, Rettungsdienst, Notfall, Qualitative Methoden, Handlungsempfehlungen, Nursing home, Paramedics, Emergency, Qualitative methods, Recommendations of action

## Abstract

**Hintergrund:**

Notfallsituationen in Pflegeeinrichtungen führen zu einer steigenden Zahl von Rettungsdiensteinsätzen und Krankenhauszuweisungen, die häufig als vermeidbar eingeordnet werden und oft nicht den Behandlungswünschen der Bewohner entsprechen. Der Umgang mit Notfallsituationen wird durch strukturelle Bedingungen, Unsicherheiten und Kommunikationsschwierigkeiten zwischen den behandelnden Akteuren beeinträchtigt.

**Ziel:**

Im Innovationsfonds-Projekt NOVELLE wird interprofessionell eine Musterhandlungsempfehlung für Pflegefachpersonen zur Strukturierung des Notfallmanagements in Pflegeeinrichtungen entwickelt.

**Material und Methoden:**

Der qualitative Forschungsprozess wurde nach der Grounded Theory als iteratives Wechselspiel zwischen Datenerhebung, -auswertung und Konzeptentwicklung organisiert. Zwischen Januar und April 2021 wurden 6 Fokusgruppeninterviews mit insgesamt 24 Pflegefachpersonen, Ärzten sowie Medizinethikern und ein Interview mit einer Medizinjuristin durchgeführt. Diese fanden als Videokonferenzen statt, wurden digital aufgezeichnet, wörtlich transkribiert und mit MAXQDA kodiert und ausgewertet.

**Ergebnisse:**

Handlungsempfehlungen zu Verbesserung und Strukturierung des Notfallmanagements lassen sich gliedern in: 1) Ersteinschätzung; 2) Assessment mit pflegefachlicher Beurteilung und Einbindung des Bewohnerwillens; 3) Organisation der Weiterversorgung. Die Bausteine enthalten Maßnahmen, Ergebnisse und Entscheidungen und lassen sich in Form eines Algorithmus anordnen.

**Diskussion:**

Handlungsempfehlungen zur Verbesserung des Notfallmanagements sollten die Kompetenzen der Pflegefachpersonen stärken, eine strukturierte Einbindung des Bewohnerwillens ermöglichen, die Kontextbedingungen berücksichtigen und bei der Kommunikation mit Weiterversorgern unterstützen.

**Zusatzmaterial online:**

Zusätzliche Informationen sind in der Online-Version dieses Artikels (10.1007/s00391-021-01958-9) enthalten.

## Abstract (en)

**Background:**

Emergencies in nursing homes lead to frequent utilization of emergency medical services and emergency department visits, which are frequently assessed as avoidable and do not comply with the patients’ wishes. Emergency management is complicated by structural conditions, uncertainty and difficulties in communication between the treating healthcare professionals.

**Objective:**

In the framework of the NOVELLE research project a model for a recommendation for action was developed in an interprofessional process to structure the emergency management in nursing homes.

**Material and methods:**

The research process was organized according to the grounded theory as a constant interplay of data collection, analysis and concept development. From January to April 2021 a total of 6 focus group interviews were conducted with 24 nurses, physicians and experts from medical ethics and 1 guideline interview with an expert from the field of medical law. All interviews were performed as video conferences. They were digitally recorded, transcribed verbatim and coded with MAXQDA software.

**Results:**

Recommendations for organizing and improving the emergency management are structured into three components: 1) initial assessment, 2) structured assessment including nursing evaluation and integration of patient treatment preferences and 3) organization of further treatment. Components include actions, results and consequences and can be arranged in the form of an algorithm.

**Discussion:**

Recommendations for improvement of emergency management should enhance the competences of nursing staff, respect patient treatment preferences, consider situational conditions and support communication with external medical and care providers.

## Hinführung zum Thema

Notfallsituationen in Pflegeeinrichtungen stellen Pflegefachpersonen vor vielfältige Herausforderungen. Dabei kommt es häufig zu Krankenhauszuweisungen, die aus medizinischer Sicht als vermeidbar eingeordnet werden, teilweise nicht mit den Behandlungswünschen der Betroffenen übereinstimmen und für diese eine zusätzliche Belastung bedeuten. Eine Ursache hierfür sind Unsicherheiten des Pflegepersonals bei der Notfallbearbeitung. In diesem Beitrag wird die multiprofessionell erarbeitete Struktur einer Musterhandlungsempfehlung zur Verbesserung des Notfallmanagements in Pflegeeinrichtungen vorgestellt.

## Hintergrund und Fragestellung

Über 800.000 Menschen in Deutschland leben in Einrichtungen der stationären Langzeitpflege [19]. Sie sind häufig von Rettungsdiensteinsätzen und Krankenhauszuweisungen betroffen, die aus medizinischer Perspektive als vermeidbar eingeschätzt werden und nicht immer mit ihrem Willen vereinbar sind [7, 9, 10, 17]. Krankenhauszuweisungen bedeuten eine Unterbrechung der kontinuierlichen pflegerischen Versorgung der zumeist vulnerablen Bewohner*innen und sind mit einem erhöhten Risiko für ein Delir, nosokomiale Infektionen, erhöhte Morbidität und einer Verschlechterung des Gesundheitszustandes verbunden [6, 12].

Eine zentrale Rolle bei der Einschätzung und Bearbeitung von Notfallsituationen in Pflegeeinrichtungen spielen Pflegefachpersonen [14]. Bei Notfällen müssen sie gleichzeitig die pflegerische Erstversorgung sicherstellen, über eine adäquate Weiterversorgung entscheiden, diese initiieren und die Versorgung des gesamten Wohnbereichs aufrechterhalten [16]. Erschwert wird die Aufgabe zusätzlich durch herausfordernde ethische, rechtliche und organisatorische Kontextbedingungen. Hierzu gehören z. B. unbekannter Bewohnerwille, unzureichende Vorausplanung, mangelhafte Personalausstattung und Qualifikation der Pflegefachpersonen, fordernde Angehörige oder eingeschränkte Erreichbarkeit von und Kommunikation mit Hausärzt*innen und ärztlichem Bereitschaftsdienst [15]. Die Angst vor rechtlichen Konsequenzen und die damit verbundenen Handlungsunsicherheiten können Pflegefachpersonen dazu veranlassen, im Zweifel den Rettungsdienst (RD) zu alarmieren [2, 3, 5].

Maßnahmen zur Verbesserung des Notfallmanagements in Pflegeeinrichtungen sollten von der Notfallwahrnehmung der erstversorgenden Pflegefachperson ausgehen und ihre Handlungsoptionen in den Blick nehmen. Allerdings überwiegt in der Literatur eine medizinische Perspektive auf das Notfallmanagement mit einem Fokus auf Symptome, Diagnosen und Krankheitsbilder [16].

Im Forschungsprojekt NOVELLE (Sektorenübergreifendes & integriertes Notfall- und Verfügungsmanagement für die letzte Lebensphase in stationärer Langzeitpflege) wird ein sektorenübergreifendes und integriertes Notfall- und Verfügungsmanagement mit und für Pflegefachpersonen in Pflegeeinrichtungen entwickelt. Ziel ist es, die Handlungssicherheit von Pflegefachpersonen im Notfallmanagement zu erhöhen und die Bewohnerautonomie zu stärken, um unnötige und nichtgewünschte Krankenhauszuweisungen zu reduzieren. Im Rahmen eines Teilprojekts wurde eine Musterhandlungsempfehlung für Pflegefachpersonen zur Strukturierung und zur Verbesserung des Notfallmanagements in Pflegeeinrichtungen entwickelt.

## Methodik

### Kontext

Die Studie wurde im Forschungsprojekt NOVELLE (FKZ: 01NVF19007; Förderung: GBA-Innovationsfonds) durchgeführt und von der Ethikkommission der Medizinischen Hochschule Hannover am 27.01.2020 als unbedenklich eingestuft (Nr. 8866_BO_K_2020).

### Studiendesign

Im Rahmen eines qualitativen Studiendesigns wurde der Forschungsprozess im Stil der Grounded Theory Methodology (GTM) als iteratives Wechselspiel zwischen Datenerhebung, -analyse und Konzeptentwicklung organisiert. Ziel war es, die Notfallbearbeitungen in Pflegeeinrichtungen aus der Perspektive von Pflegefachpersonen zu rekonstruieren, zu strukturieren und aus diesen Ergebnissen eine Musterhandlungsempfehlung für das Notfallmanagement zu entwickeln.

### Auswahl der Teilnehmenden

Die Teilnehmenden wurden gezielt aus den folgenden zwei Gruppen ausgewählt:

1. an der Notfallversorgung beteiligte Pflegefachpersonen, Pflegedienstleitungen (PDL), Einrichtungsleitungen und Ärzt*innen (Allgemeinmedizin, Geriatrie, Onkologie, Palliativmedizin, Rettungsmedizin),
2. am Forschungsprojekt beteiligte Wissenschaftler*innen mit medizinrechtlicher und -ethischer Expertise in Fragen der Notfallversorgung in Pflegeeinrichtungen.

Die Teilnehmenden aus Gruppe 1 wurden zwischen Oktober 2020 und Januar 2021 in der Interventionsregion im Sinne des „purposive sampling“ rekrutiert. Es handelte sich dabei zunächst um Mitglieder eines regionalen Arbeitskreises zur Verbesserung der Versorgungssituation in Pflegeeinrichtungen sowie um Mitarbeitende von Pflegeeinrichtungen, die am Gesamtprojekt beteiligt sind. Alle Teilnehmenden dieser Gruppe erhielten eine Aufwandsentschädigung.

Die Teilnehmenden aus Gruppe 2 waren Mitarbeitende der verschiedenen NOVELLE-Projektpartner mit Expertise in den relevanten Themengebieten. Sie wurden angefragt, um spezifische interdisziplinäre Fragestellungen zu klären.

Alle Teilnehmenden erklärten ihr schriftliches Einverständnis zur Studienteilnahme und erhielten eine ausführliche Studieninformation, Datenschutzerklärung und eine Anleitung zur Videokonferenz.

### Forschungsprozess

Zwischen Januar und April 2021 wurden 6 Fokusgruppeninterviews und ein Einzelinterview mit insgesamt 25 Teilnehmenden in unterschiedlicher Zusammensetzung durchgeführt. Alle Termine fanden als Videokonferenzen über die Software BigBlueButton statt. Sie dauerten zwischen 92 und 122 min, wurden digital aufgezeichnet, wörtlich transkribiert und mithilfe der Software MAXQDA20 (Software für qualitative Datenanalyse) kodiert und ausgewertet. In Anlehnung an den Forschungsstil der GTM wurden die Zusammensetzung der Gruppen und die inhaltliche Gestaltung der Sitzungen im Forschungsprozess (Tab. 1) auf Basis erster Zwischenergebnisse und theoretischer Überlegungen variiert [4].

**Table 1:** 

Erhebungsmethode	Inhaltlicher Fokus	Anzahl und Berufsgruppenzugehörigkeit
Fokusgruppe (FG)	Rekonstruktion und Weiterentwicklung der Notfallbearbeitung „Sturz“	3 Pflegefachpersonen und PDL
FG	Rekonstruktion und Weiterentwicklung der Notfallbearbeitung „entgleiste Vitalwerte“	4 Pflegefachpersonen und PDL
FG	Weiterentwicklung des Bausteins „Organisation der Weiterversorgung“	1 Leitungspersonal aus Pflegeeinrichtungen 3 Ärzt*innen (Allgemein- und Palliativmedizin)
FG	Weiterentwicklung des Bausteins „Assessment“	1 Leitungspersonal aus Pflegeeinrichtung 2 Medizinethiker*innen
FG	Rekonstruktion und Weiterentwicklung der Notfallbearbeitung „Schmerz“	5 Pflegefachpersonen und PDL
FG	Rekonstruktion und Weiterentwicklung der Notfallbearbeitung „Luftnot“	5 Pflegefachpersonen und PDL
Einzelinterview	Klärung rechtlicher Fragen	1 Medizinjuristin

**Figure 1:**
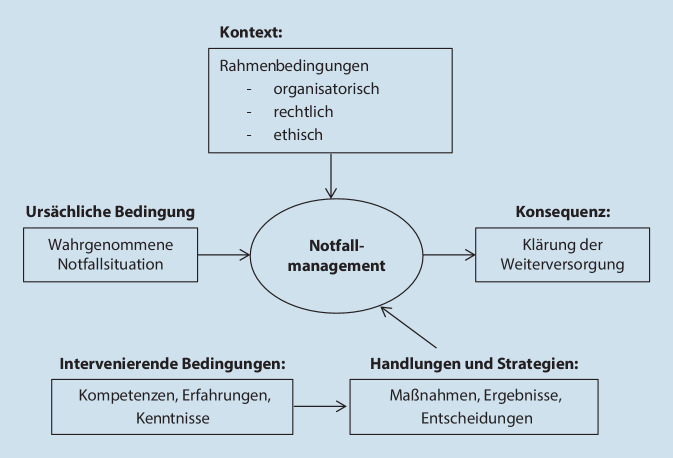

**Figure 2:**
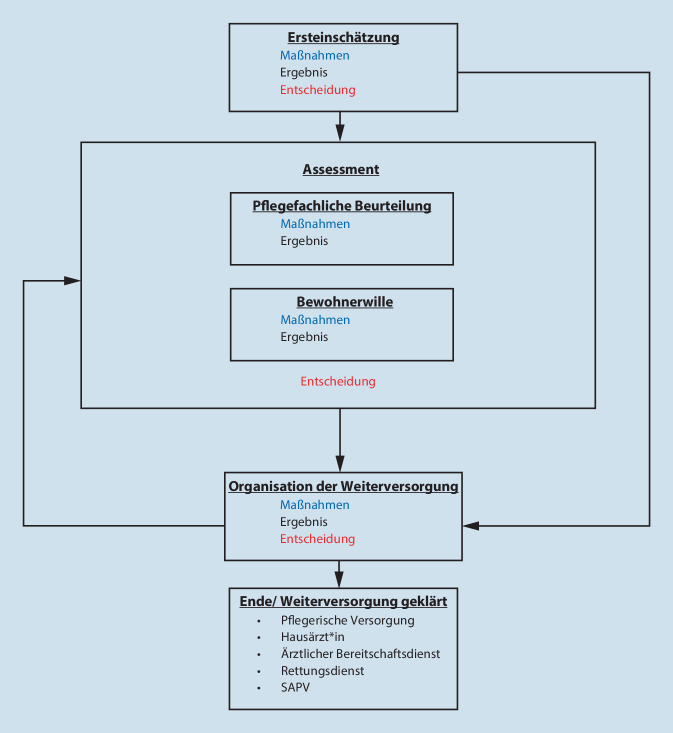

Erst- und Letztautor*in kodierten zunächst unabhängig voneinander und führten die offenen Kodes anschließend zusammen. Im Forschungsteam wurden anschließend die Kodes miteinander in Beziehung gesetzt und mithilfe des paradigmatischen Modells axial kodiert (Abb. 1; [18]). Durch permanentes Vergleichen der Kodes wurde herausgearbeitet, dass in allen Notfallsituationen ähnliche Bearbeitungsschritte durchlaufen werden. Im Rahmen der Konzeptentwicklung der Musterhandlungsempfehlung wurden diese Bearbeitungsschritte in Form eines Ablaufdiagramms geordnet und miteinander in Beziehung gesetzt (Abb. 2).

Mit zwei abschließenden Fokusgruppen mit Pflegefachpersonen zu den Notfallsituationen „Schmerzen“ und „Luftnot“ wurde eine theoretische Sättigung erreicht und die Datenerhebung und -auswertung beendet.

## Ergebnisse

Das zentrale Ergebnis der Datenanalyse ist eine Musterhandlungsempfehlung für Pflegefachpersonen zur Strukturierung des Notfallmanagements in Pflegeeinrichtungen. Diese besteht aus drei Bausteinen: 1) Ersteinschätzung; 2) Assessment; 3) Organisation der Weiterversorgung. Für jeden Baustein können Maßnahmen, Ergebnisse und Entscheidungen identifiziert werden. Die Bausteine lassen sich in Form eines Algorithmus anordnen (Abb. 2).

### Ersteinschätzung

Die Ersteinschätzung einer Notfallsituation durch Pflegefachpersonen bildet die Ausgangslage für die weitere Notfallbearbeitung. Sie findet auf Grundlage des ersten Eindrucks und der spontan verfügbaren Kenntnisse der Pflegefachperson über den/die Bewohner*in statt.

Sehr dringlich wäre zum Beispiel Bewusstlosigkeit, Schaum vorm Mund oder Augen raus, ich weiß es nicht. Dann muss man sehr dringend Kontakt zur Rettungsleitstelle [aufnehmen]. (FG2P5: 23)

Infolge der Ersteinschätzung wird die Entscheidung getroffen, ob eine umfassendere Beurteilung durchgeführt oder der Rettungsdienst unmittelbar alarmiert wird. Zentrale Kriterien für diese Entscheidung sind dabei die eingeschätzte Schwere und die Dringlichkeit der Notfallsituation.

### Assessment^1^

Assessment bezeichnet die Handlungen und Strategien von Pflegefachpersonen zu strukturierter Erfassung und Reflexion von Notfallsituationen und Wahl einer Weiterversorgung. Im Rahmen des Assessments finden eine pflegefachliche Beurteilung sowie eine Einbindung des aktiv ermittelten Bewohnerwillens statt.

Zur pflegefachlichen Beurteilung der Notfallsituation gehören je nach spezifischer Situation zahlreiche Maßnahmen, wie die Rekonstruktion des Notfallgeschehens, die Befragung der Person und die körperliche Untersuchung.

Was wir haben in unserer Dokumentation ist ein Sturzprotokoll, wo dann die Vitalwerte eingetragen werden, wo der Bodycheck durchgeführt wird, wo der Sturzhergang beschrieben wird oder wie wir die Person vorgefunden haben, wenn wir beim Sturz nicht dabei waren. (FG1P3: 164)

Nach der pflegefachlichen Beurteilung der Notfallsituation wird das weitere Vorgehen mit den Behandlungswünschen des/der Bewohner*in abgestimmt. Dabei geht es sowohl um die Frage, ob die Einwilligungsfähigkeit vorliegt und die Behandlungswünsche erörtert werden können, als auch um die Frage, inwieweit die Behandlungswünsche mit der pflegefachlichen Beurteilung korrespondieren.

Erstens ist […] der Bewohner einwilligungsfähig. Dann weiß ich ja überhaupt, muss ich dann den Bevollmächtigten informieren darüber oder muss ich den Bevollmächtigten in der Entscheidungssituation miteinbeziehen? (FG3W2: 47)

Stimmen die pflegefachliche Beurteilung und die Behandlungswünsche der Bewohner*innen nicht überein, kann es zu kritischen Situationen kommen, in denen eine Entscheidung gegen den Bewohnerwillen getroffen wird:

Also da würde ich tatsächlich den Rettungsdienst, gerade wenn mir das jetzt nachts oder so passiert, da würde ich […] auf den Bewohner gut einreden und sagen: „Bitte, bitte, lassen Sie mich den Rettungsdienst rufen.“ Ich möchte in diesem Fall wirklich jetzt hier nicht die Verantwortung haben. (FG3P7: 78)

Die gesammelten Informationen sollen zudem den Weiterversorger bei der Entscheidungsfindung unterstützen:

Also ich finde es tatsächlich auch sinnvoll, dass man direkt schon ein paar Werte mitteilen kann, […] damit der Arzt dann aufgrund dieser Vitalwerte auch direkt entscheiden kann, was gemacht werden soll. (FG2P3: 120)

### Organisation der Weiterversorgung

Die Organisation der Weiterversorgung umfasst den Prozess, in dem die Umsetzung abgestimmt und organisiert wird. Auf Basis des vorhergehenden Assessments wird entschieden, ob eine interne Weiterversorgung in der Pflegeeinrichtung möglich ist oder externe Akteure hinzugezogen werden müssen.

Ja, also wenn […] ich die Rettungsleitstelle anrufe, dann schildere ich ja schon mal was ist, was liegt vor, was habe ich für Vitalwerte, wie ist es, wie schätze ich das hier vor Ort ein. (FG2P2: 129)

Dabei wird abgestimmt, ob die geplante Weiterversorgung in einem gewählten Zeitfenster möglich ist, und überprüft, ob das präferierte Vorgehen unter Berücksichtigung der Kontextbedingungen umsetzbar ist. Zu den Kontextbedingungen zählen z. B. Angehörige, die Druck auf Pflegefachpersonen ausüben oder mangelnde Personalbesetzung zur Gewährleistung der Versorgung des Wohnbereichs. Zudem gestaltet sich die Kontaktaufnahme mit Hausärzt*innen und ärztlichem Bereitschaftsdienst z. T. herausfordernd.

Ich habe tatsächlich auch gemerkt, dass man, wenn ich im Spätdienst dann einen Arzt erreichen muss und auch da teilweise 20, 25 min in der Warteschleife hänge, in der Zeit Bewohner versorgt werden müssen, und wenn man dann teilweise sogar zwei Notfälle auf einem Bereich hat, dann wird [es] schon echt ganz schön brisant. (FG1P3:134)

Ist die geplante Weiterversorgung aufgrund der Kontextbedingungen nicht umsetzbar, oder ergibt sich aus der Abstimmung mit dem Versorgungsakteur eine andere Einschätzung der Situation, kann das geplante Vorgehen revidiert werden. Ein Abschluss des Prozesses ist erreicht, wenn die Weiterversorgung für den vorgesehenen Zeitraum verbindlich geklärt ist.

## Diskussion

Die Notfallbearbeitung in Pflegeeinrichtungen durch Pflegefachpersonen lässt sich in 3 Phasen untergliedern, die als Bausteine einer Muster-Handlungsempfehlung angeordnet werden können: 1) Ersteinschätzung, 2) Assessment und 3) Organisation der Weiterversorgung. Die Bausteine bestehen jeweils aus Maßnahmen, Ergebnissen und Entscheidungen und führen in der Konsequenz zur Einbindung einer geeigneten Weiterversorgung. Die Musterhandlungsempfehlung soll dazu dienen, die Notfallbearbeitung in Pflegeeinrichtungen durch Pflegefachpersonen zu strukturieren.

Das Notfallmanagement in Pflegeeinrichtungen ist in erster Linie eine Aufgabe der dort tätigen Pflegefachpersonen, die mit zahlreichen Herausforderungen konfrontiert sind. Zwar existieren bereits Handreichungen, Standards und Empfehlungen zur Notfallversorgung in Pflegeeinrichtungen (z. B. [1, 8, 11]). Allerdings fokussieren diese primär auf die praktische Erstversorgung, geben kaum Hinweise auf die Einbindung des Bewohnerwillens, sind selten empirisch fundiert und enthalten nur wenige Informationen für die Organisation eines Weiterversorgers. Die hier entwickelte Musterhandlungsempfehlung bietet auf Basis empirischer Ergebnisse einen umfassenderen Rahmen zur Strukturierung des Notfallmanagements und berücksichtigt die Organisation einer adäquaten Weiterversorgung. Sie kann Pflegefachpersonen eine Hilfestellung für die gezielte Reflexion, Entscheidungsfindung und ggf. auch Dokumentation der relevanten Handlungsschritte bieten.

Seit 2017 wird das Advance Care Planning (ACP) krankenkassenfinanziert auch in deutschen Pflegeeinrichtungen implementiert, um die Patientenautonomie in der letzten Lebensphase zu verbessern [20]. Allerdings zeigt sich im internationalen Kontext, dass die Behandlungswünsche der Bewohner*innen in Akutsituationen trotz ACP häufig unberücksichtigt bleiben [13]. Mit der in dieser Studie entwickelten Musterhandlungsempfehlung können die Behandlungswünsche regelhaft in die systematische Notfallbearbeitung integriert werden. Die Benennung konkreter Maßnahmen zu ihrer Ermittlung und die Verknüpfung der Ergebnisse mit Konsequenzen sollen dazu beitragen, die Patientenautonomie häufiger auch in Akutsituationen zu wahren.

Das Notfallmanagement in Pflegeeinrichtungen findet oft unter herausfordernden Rahmenbedingungen statt, die eine pflegefachlich präferierte Weiterversorgung im Einklang mit den Behandlungswünschen der Bewohner*innen erschweren [2, 15]. Die Musterhandlungsempfehlung berücksichtigt im Baustein „Organisation der Weiterversorgung“ diese komplexe Versorgungsrealität und lässt die Kontextbedingungen in die Wahl der Weiterversorgung einfließen. Sie kann Pflegefachpersonen davor schützen, strukturelle und organisatorische Defizite als individuelle Unzulänglichkeiten zu interpretieren, indem beispielsweise die Einbindung des Rettungsdienstes unter ungünstigen Kontextbedingungen als nachvollziehbare Alternative aufgezeigt wird [16].

Pflegefachpersonen sehen sich bei der Notfallbearbeitung zudem mit rechtlichen und ethischen Unsicherheiten konfrontiert, die eine Alarmierung des Rettungsdienstes zur Folge haben können [2, 3]. Die vorgelegte Musterhandlungsempfehlung wurde gemeinsam mit Ärzt*innen, Pflegefachpersonen und -leitungspersonal sowie Wissenschaftler*innen entwickelt. Sie kann dadurch dazu beitragen, die Handlungssicherheit von Pflegefachpersonen in Notfallsituationen zu verbessern.

### Limitationen

Die Forschungsfrage fokussierte auf die Notfallwahrnehmung und -bearbeitung von Pflegefachpersonen, obwohl auch zahlreiche Pflegehilfspersonen und weitere Berufsgruppen in den Einrichtungen tätig sind. Eine Übertragbarkeit auf diese Berufsgruppen ist nicht ohne Weiteres möglich.

Die Gewinnung der Pflegefachpersonen und Ärzt*innen erfolgte im Rahmen des Purposive sampling. Hierzu wurden gezielt Personen kontaktiert, die sich bereits am NOVELLE-Projekt beteiligen. Eine positive Selektion von besonders engagierten Praxisteilnehmenden kann nicht ausgeschlossen werden.

## Ausblick

Die vorliegende Struktur einer Musterhandlungsempfehlung bildet ein theoretisches Modell für die Strukturierung der Notfallbearbeitung in Pflegeeinrichtungen durch Pflegefachpersonen und wird im weiteren Projektverlauf für konkrete Notfallsituationen spezifiziert und erweitert.

## Fazit für die Praxis (Wissenschaft und/oder Versorgung) in einem Satz

Interprofessionell und interdisziplinär erarbeitete Handlungsempfehlungen können die Grundlage für eine Stärkung der Handlungssicherheit von Pflegefachpersonen und der Patientenautonomie beim Notfallmanagement in Pflegeeinrichtungen sein.

## Supplementary Information





## Footnotes

1 Hier ist nicht das geriatrische Assessment oder das Pflegeassessment gemeint, sondern die möglichst strukturierte Informationsgewinnung und -reflexion unter zeitkritischen Bedingungen einer Notfallsituation.
