# Supplementary material for: Structuring emergency management in nursing homes: Results of interprofessional focus group interviews
Source: Z Gerontol Geriatr. 2021 Aug 13;55(7):569–74. [Article in German] doi: 10.1007/s00391-021-01958-9 (PMC9587113; doi:10.1007/s00391-021-01958-9)
Supplement: Supplementary file 1 [file 391_2021_1958_MOESM1_ESM.docx]

**Online-Supplement**

**Ergänzungen zum Forschungsprozess**

Wir entschieden uns zunächst für die Rekonstruktion der Notfallbearbeitung aus der Perspektive von Pflegefachpersonen anhand der Notfallsituationen „Sturz“ und „entgleiste Vitalwerte bei Routineuntersuchung“. Auf Basis der Literaturrecherche und vorangehender Projektergebnisse [15, 16] wurde ein Leitfaden erstellt, der zunächst anhand eines Fallbeispiels den Prozess der Notfallbearbeitung thematisierte und anschließend detailliertere Beschreibungen zu konkreten Ausprägungen der Notfallsituationen (z.B. schlechtere Erreichbarkeit des Hausarztes, fordernde Angehörige) evozierte. Leitend hierfür war die Annahme, dass sich die beiden Notfallsituationen in Bezug auf die Dringlichkeit (Sturz: Akutes Ereignis; entgleiste Vitalwerte bei Routineuntersuchung: schleichender Prozess) in der Notfallbearbeitung voneinander unterscheiden und dadurch eine große Ergebnisvarianz erzeugen.

Nach den ersten beiden Fokusgruppen wurde deutlich, dass zentrale Fragen zur Organisation der Weiterversorgung und zur Einbindung des Bewohnerwillens für die Entwicklung der Muster-Handlungsempfehlung offengeblieben sind. Wir führten daher zwei weitere Fokusgruppen und ein Einzelinterview mit den ärztlichen Weiterversorgern und Wissenschaftler*innen aus Ethik und Recht mit angepassten Leitfäden durch. Dabei stellten wir zunächst die Muster-Handlungsempfehlung vor, präsentierten Fallgeschichten aus den vorausgegangenen Fokusgruppen und generierten durch offene, immanente und exmanente Nachfragen Narrationen zu den relevanten Themen.
